# Rash caused by lurasidone in old chinese patient with bipolar disorder: case-based review

**DOI:** 10.1186/s12888-024-05668-5

**Published:** 2024-07-08

**Authors:** Wenjuan Yang, Danhong Hu, Bei Zheng, Bing Han, Pingping Feng, Yongcan Zhou, Weixin Wang, Gonghua Li, Meiling Zhang

**Affiliations:** 1 Department of Pharmacy, Tongde Hospital of ZheJiang Province, Hangzhou, Zhejiang People’s Republic of China; 2 Zhejiang Academy of Traditional Chinese Medicine, Hangzhou, Zhejiang People’s Republic of China; 3 Zhejiang Mental Health Center, Hangzhou, Zhejiang People’s Republic of China; 4 Department of pharmacy, ZheJiang Hospital of ZheJiang Province, Hangzhou, Zhejiang People’s Republic of China; 5 Department of Psychiatry, Tongde Hospital of ZheJiang Province, Hangzhou, Zhejiang People’s Republic of China

**Keywords:** Rash, Lurasidone, Dose increasing, Adverse drug reaction

## Abstract

**Background:**

Rash is one of common adverse drug reaction and which have been reported in typical and atypical antipsychotics. Reports of lurasidone induced skin reactions are sparse. In this study, we report a case of rash caused by lurasidone.

**Case presentation:**

A 63-year-old man with bipolar disorder (BD) who is treated by lurasidone. However, the patient presents a rash all over after lurasidone dose increasing from 40 mg/day to 60 mg/day. With the diagnosis of drug induced rash, lurasidone was discontinued, and the rash complete disappears within 2 weeks. In addition, all case reports about antipsychotics associated rash were reviewed by searching English and Chinese database including Pubmed, Embase, Cochrane Library, CNKI and Wanfang database. A total of 139 articles contained 172 patients were included in our study. The literature review and our case suggest that the cutaneous adverse events caused by antipsychotic drugs should not be ignored, particularly for the patient who was first use or at dose increasing of antipsychotic.

**Conclusions:**

In conclusion, we report a case of lurasidone related rash and review rash caused by antipsychotics. Psychiatrists should be alert to the possibility of the rash caused by antipsychotics, especially the patient was first use of antipsychotics or the antipsychotic dose was increasing.

**Supplementary Information:**

The online version contains supplementary material available at 10.1186/s12888-024-05668-5.

## Background

Bipolar disorder (BD) is a kind of chronic debilitating mental disorder characterized by recurrent episodes of mania/hypomania, depression and euthymia [1]. The main characteristic of BD is recurrent mood episodes, which have prevalence of between 1% and 2% [2, 3]. A systematic analysis reviewed that BD accounted for 24.8 million estimated cases in 1990 and 39.5 million cases in 2019, corresponding to an increase of 59.3% approximately between 1990 and 2019 [4]. BD significantly reduce psychosocial functioning of the patients, and is associated with approximately 10-20 years of potential life lost [5]. In China, the incidences of BD increased by 47.74%, from 3.06 million in 1990 to 4.53 million in 2017, and the disability-adjusted life years (DALYs) increased by 54.4%, from 6.02 million in 1990 to 9.29 million in 2017 [6]. The chronic episodic course of the BD affects not only patients’ interpersonal relationships but also occupational functioning, even lead to severe outcomes, including death by suicide [7]. Study showed that BD is a major risk factor for suicide, and which is current in up to 14% of all suicide deaths. The suicide rates in BD patients up to 20 times higher than the general population [8].

It is acknowledged that mood stabilizers (MSs) are the mainly therapeutic drugs used to treat BD in the acute phase or for maintenance therapy to prevent relapse [9]. MS was used on manic and/or depressive symptoms during an acute episode. In addition, MS was also used in the prevention of manic and/or depressive recurrences [10]. Lithium, one of first-generation of mood stabilizers (FGMSs) for BD, is considered the best treatment for BD and works outstandingly well in about 30% of patients [11]. Additionally, several antiepileptic drugs (AED) like valproate and carbamazepine, two other FGMSs, were demonstrated efficacy in the treatment of BD. Second-generation mood stabilizers (SGMSs) including second-generation antipsychotics (SGA) and lamotrigine were also considered as MSs and approved for BD treatment [12]. A cross-sectional survey study reviewed that mood-stabilizing AED, SGA, and antidepressants were the most prescribed medications in patients with bipolar disorder [13].

Lurasidone, a new atypical antipsychotic, which blocks dopamine D2 and serotonin 5-hydroxy-tryptamine (5-HT) 2 A receptors and affects other serotoninergic and noradrenergic receptors [14]. Lurasidone was approved by the FDA in 2010 for use in schizophrenia, and for approved by the Food and Drug Administration (FDA) in 2013 for treatment in bipolar depression [15]. In China, lurasidone was approved in 2019 by China’s National Medical Products Administration (NMPA) for the treatment of schizophrenia in adults [16]. Now, lurasidone was widespread used in schizophrenia and bipolar depression [17, 18]. Most study showed that lurasidone was effective in improving symptoms of depression of bipolar disorder type I (BD-I). However, recent study reviewed that lurasidone is a potential option for treating depressive episodes in bipolar disorder type II (BD II) and other specified bipolar and related disorders (OSBD) [19]. Additionally, study reviewed that lurasidone monotherapy significantly improved depressive symptoms in patients with non-rapid cycling bipolar depression [20]. Furthermore, a post hoc analysis reviewed that lurasidone was better than placebo in reducing psychic and somatic anxiety in the short-term treatment of bipolar depression [21]. Moreover, lurasidone have advantage in first-episode psychosis with predominant depressive symptoms [22].

Common adverse drug reaction of lurasidone include nausea, vomiting, drowsiness, and akathisia[23]. Lurasidone skin rashes are rare. In present study, we report a patient with BD experienced rash after lurasidone treatment. To our acknowledge, this is the first study to report rash caused by suspected lurasidone in Chinese patient.

## Case presentation

A 63-year-old man weighing 60 kg was presented to the inpatient psychiatry unit on May 12, 2023, with symptoms of negative, fear and anxiety lasted for 1 month. He was diagnosed with BD and anxiety-depressive state. Diagnostic and Statistical Manual of Mental Disorders-5 (DSM-5) diagnostic criteria was used to assess BD in our case. The patient has history of mental disorders over 30 years, and he has a medical history of hypertension, diabetes and coronary atherosclerotic cardiopathy. The patient has been hospitalized for BD in our hospital from October 15 to December 17, 2022, sodium valproate (500 mg BID), aripiprazole (10 mg QN) and lorazepam (0.5 mg QN) were used duration of hospital stay and subsequent discharge. The other past medications were venlafaxine, citalopram, mirtazapine, sertraline, lamotrigine, escitalopram, and olanzapine. The patient had no known allergies at the time of admission. The patient’s current medication regimen consisted of acarbose (200 mg TID), irbesartan (150 mg QD), rosuvastatin (10 mg QN), lurasidone (40 mg QD), lorazepam (0.5 mg TID) and lithium carbonate (0.3 g TID).

On the 2th day of hospitalization, sodium valproate (500 mg BID) was continued, creatinine of the patient was 109µmol/L and with no other abnormalities. On the 4th day of hospitalization, the dosage of lurasidone was increased to 60 mg QD due to the symptoms were not alleviated. The following day, several rashes were appeared in his arm. On the 6th day of hospitalization, the patient developed a maculopapular rash over the whole body and vomiting. The body temperature was 36.6℃. Neutrophile granulocyte (%), lymphocyte (%), eosinophilic granulocyte (%), monocyte (%), white blood cell count, eosinophilic granulocyte count and neutrophil count were 80.9%, 12.2%, 3.8%, 2.9%, 8.9*10^9^/L, 0.34*10^9^/L and 7.2*10^9^/L, respectively. The levels of alanine aminotransferase (ALT) and aspartate aminotransferase (AST) were 16U/L and 19 U/L, respectively. However, his creatinine was increased to 112µmol/L. Thus, lithium carbonate was reduced from 0.3 g TID to 0.3 g BID, lorazepam was reduced from 0.5 mg TID to 0.5 mg BID. In addition, sodium valproate was discontinued on that day because of the possibility of causing a rash. Unfortunately, the next day, the rashes were not improvement, and the patient complained of nausea and vomiting. Therefore, lurasidone was discontinued on the 7th day of hospitalization. Meanwhile, loratadine (10 mg QN for 3days) and prednisone (5 mg TID for 3days) were used. On the 8th day of hospitalization, lorazepam and lithium carbonate were increased to 0.5 mg TID and to 0.3 g TID, respectively. On the 9th day of hospitalization, the dose of lithium carbonate was increased to 0.3 g TID. On the 10th day of hospitalization, the rashes were improvement. On the 15th day of hospitalization, quetiapine (25 mg QN) was used to improve insomnia, and lorazepam was gradually discontinued. The patient’s mental symptoms were well controlled. On the 25th day of hospitalization, aripiprazole was used due to the patient repeated pestering for drug reduction. Finally, the patient was treated with lithium carbonate combined with aripiprazole, and his symptoms improved. He was eventually discharged from the hospital on the 85th day of hospitalization.

## Literature review

PubMed, Embase, Cochrane Library, CNKI, and Wanfang Database were searched for the period of database inception to August 2, 2023. The main search terms including “skin rash”, “rash skin”, “rash, exanthem”, “antipsychotic agents”, “antipsychotics”, “antipsychotic drugs”, “atypical antipsychotics”, “typical antipsychotics”, “first-generation antipsychotics” and “second-generation antipsychotics”. Inclusion criteria were: 1) case or cases which described of rash; 2) antipsychotics were used; Exclusion criteria were: 1) the patient have not been treated by antipsychotics; 2) data were not available; 3) review, book chapters, and conference report.

## Discussion and conclusions

In our case, we report an old male patient with BD who developed rash after increasing dose of lurasidone from 40 mg/day to 60 mg/day. There was no history of drug allergy in the patient. No previous use of lurasidone. In the case, sodium valproate and lurasidone were discontinued when rash was happened. However, the patient has been hospitalized in our hospital from October 15 to December 17, 2022, and sodium valproate (500 mg BID) was prescribed during the hospital stay. Moreover, he took sodium valproate intermittently after discharge and no adverse drug reaction of sodium valproate was reported. Additionally, sodium valproate was discontinued the day when rash appeared, however, the symptoms did not improved. Thus, lurasidone, which was prescribed in the patient first time, worth considering for the only reason of drug eruption. The association of rash and lurasidone was “probable” based on the Naranjo adverse drug reaction probability scale (Table 1)[24].

**Table 1:** Naranjo ADR probability scale

	Question			Answer	Score
1	Are there previous conclusive reports on this reaction?			Yes	1
2	Did the adverse event appear after the suspected drug was administered?			Yes	2
3	Did the adverse reaction improve when the drug was discontinued or a specific antagonist was administered?			Yes	1
4	Did the adverse reaction reappear when the drug was readministered?			Do not know	0
5	Are there alternative causes (other than the drug) that could on their own have caused the reaction?			Yes	-1
6	Did the reaction reappear when a placebo was given?			Do not know	0
7	Was the drug detected in the blood (or other fluids) in concentrations known to be toxic?			No	0
8	Was the reaction more severe when the dose was increased, or less severe when the dose was decreased?			Yes	1
9	Did the patient have a similar reaction to the same or similar drug in any previous exposure?			No	0
10	Was the adverse event confirmed by any objective evidence?			Yes	1
Total Score				5	
**Probability**	< 0: Doubtful	1–4: Possible	5–8: Probable	> 9: Definite	

It is well known that weight gain and metabolic syndrome are the common adverse drug reactions of atypical antipsychotics. Compared with other atypical antipsychotics, lurasidone offers a more safety advantage. Study showed that compared to olanzapine, quetiapine and risperidone, lurasidone has a decreased risk of metabolic side effects such as hypercholesterolemia, hyperlipidemia, hyperglycemia, and weight gain [25]. Recently, study which base on the FDA Adverse Event Reporting System (FAERS) database (from 4th quarter of 2010 to the 3rd quarter of 2023) exhibited that psychotic system-related adverse events (AEs) (e.g., insomnia, mania, agitation, etc.) and extrapyramidal symptoms were the main adverse events of lurasidone, which about 28.56%. The second type of AEs were all kinds of neurological diseases, which about 16.39%. Incidence of diseases of the skin and subcutaneous tissue was 2.64% [26]. A previous study showed that an old white woman with schizoaffective disorder who received lurasidone 20 mg daily, and she was experience a rash 8 days after the dose of lurasidone was increased to 40 mg daily [27]. Our case was similar to this case, and they were all experience a rash when dose of lurasidone was increasing. Furthermore, the molecular structure of lurasidone is similar to perospirone which researched by Dainippon Sumitomo Pharma in 2001 [28]. A previous case of rash caused by perospirone has been reported, and the rash expands after increasing dose [29]. Thus, the possible reason of lurasidone induced rash we think was dose-dependent lurasidone associated with rash. Additionally, study exhibited that some drugs metabolized to reactive sulfate metabolites which are responsible for skin rashes [30]. Lurasidone belongs to the class of benzothiazol derivatives, and we speculate that the rashes were caused by the possible reactive sulfate metabolites. Moreover, concomitant medications and age should also be considered.

In our case, Naranjo ADR Probability Scale was used to determine drug eruptions. Naranjo ADR Probability Scale, which consists of ten ADR-related medical questions with a predetermined score, is mainly used to evaluate and determine the causal relationship between drug use and ADR, and is conducive to the identification and discovery of adverse drug reactions, including rare adverse drug reactions [31]. Thus, it is validity for determine drug eruptions. In our case, the rash had a maculopapular appearance in limbs, chest and back after increasing lurasidone dose, and thus maculopapular drug eruption was considered by Naranjo ADR Probability Scale. However, dermatologist was not consulted. Study showed a case of drug reaction with eosinophilia and systemic symptoms (DRESS) syndrome with possible neosensitization to lurasidone in a patient with BD. The patient was developed DRESS syndrome after risperidone and valproate treatment, and subsequent rash recurrence when lurasidone trial [32]. Although the cutaneous eruption has also been described as a maculopapular or erythematous rash in case reports of DRESS syndrome [33]. However, DRESS syndrome was rule outed in our case due to the no abnormalities of body temperature, white blood cell count, eosinophilic granulocyte (%), and eosinophilic granulocyte count.

**Fig. 1:**
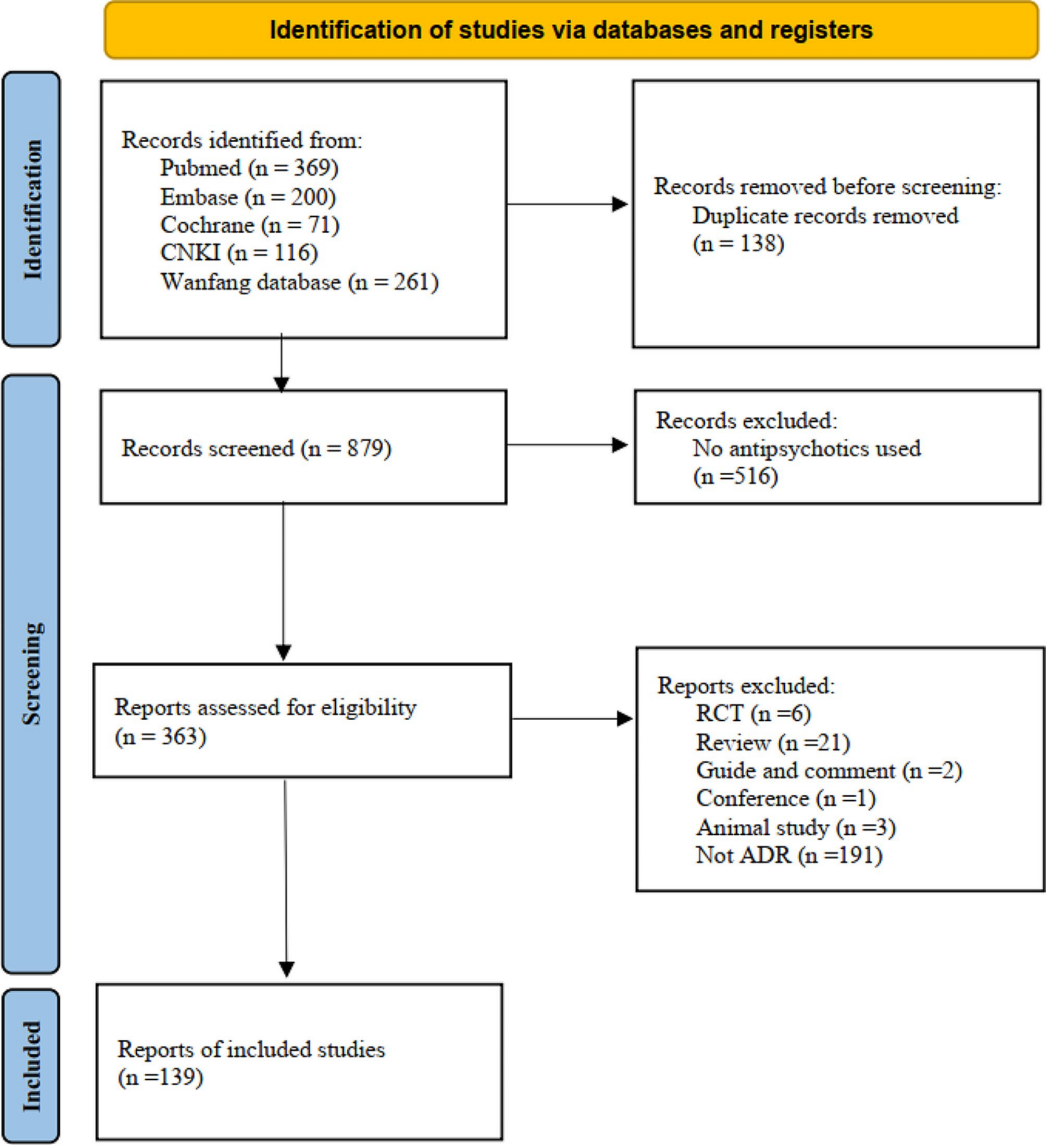
PRISMA flow diagram of the study selection process

Based on the current literature, out of the 1017 studies identified in our search, 139 met the inclusion criteria. A flow-chart of the search and screening process can be found in Fig. 1. 172 patients aged between 10 months and 78 years old were included (Table 2). 16 antipsychotics in total were included in our study. From our result, clozapine, risperidone and chlorpromazine were the first top three antipsychotics which caused rash. Moreover, rash developed after treated with antipsychotics could in different forms, including exfoliative dermatitis, DRESS syndrome, acne, symmetrical drug-related intertriginous and flexural exanthema (SDRIFE), etc. The rash could appeared after initial use, and which could also happened after increasing dose of antipsychotics [34]. Furthermore, most of rash in our review was happen within 10 days, and three cases of rash were occurred 1 years after antipsychotics used.

**Table 2:** Demographic and clinical features of the previously published cases of rash associated with antipsychotics

Variables	Total (*N* = 172)	Total (%)	Mean
Sex			
Female	83	48.26	
Male	89	51.74	
**Age**			34.29 (*N* = 163)
≥ 60 years	13	7.56	
≥ 18 years, < 60 years	138	80.23	
< 18 years	12	6.98	
ND	9	5.23	
**Second-generation antipsychotics**			
Amisulpride	3	1.74	
Aripiprazole	12	6.98	
Clozapine	42	24.42	
Lurasidone	2	1.16	
Olanzapine	14	8.14	
Paliperidone	3	1.74	
Perospirone	1	0.58	
Quetiapine	14	8.14	
Risperidone	25	14.53	
Ziprasidone	2	1.16	
**First-generation antipsychotics**			
Chlorpromazine	25	14.53	
Haloperidol	13	7.56	
Perphenazine	3	1.74	
Sulpiride	13	7.56	
Tiapride	1	0.58	
Zuclopenthixol	1	0.58	

ND, information not described

However, there several limitations in our report. Firstly, the number of cases in our study was small. Secondly, blood lurasidone concentration was not tested in the case when dose increased. Thus, further studies are warranted to determine the dosage and timing of onset of lurasidone induced rash when increased dose of lurasidone.

## Conclusion

In conclusion, we report a case of lurasidone related rash and review rash caused by antipsychotics. It is less common at rash induced by antipsychotics compared with metabolic syndrome and psychotic system-related adverse events. However, it can be very bothersome and lead to the termination of treatment. Thus, psychiatrists should be alert to the possibility of the rash caused by antipsychotics, especially the patient was first use of antipsychotics or the antipsychotic dose was increasing.

### Electronic supplementary material

Below is the link to the electronic supplementary material.

Supplementary Material 1

## Data Availability

The data supporting the findings of this report are available from the corresponding author upon reasonable request.

## Abbreviations

BD: Bipolar disorder
DALYs: Disability-adjusted life years
MS: Mood stabilizer
FGMSs: First-generation of mood stabilizers
AED: Antiepileptic drug
SGMSs: Second-generation mood stabilizers
SGA: Second-generation antipsychotic
NMPA: National Medical Products Administration
FDA: Food and Drug Administration
FAERS: FDA Adverse Event Reporting System
AEs: Adverse events
DRESS: Drug reaction with eosinophilia and systemic symptoms
SDRIFE: Symmetrical drug-related intertriginous and flexural exanthema
