# Supplementary material for: Rash caused by lurasidone in old chinese patient with bipolar disorder: case-based review
Source: BMC Psychiatry. 2024 Jul 8;24:491. doi: 10.1186/s12888-024-05668-5 (PMC11229191; doi:10.1186/s12888-024-05668-5)
Supplement: Supplementary file 1 — Supplementary Material 1 [file 12888_2024_5668_MOESM1_ESM.docx]

Table S1: Included studies.

| No. | **Author and Year** | **Country** | **Article type** | **Number of cases** | **Sex** | **Age** | **Diagnose** | **Psychiatric Drug** | **The time to develop cutaneous adverse drug reactions** | **Drug combination** | **Cutaneous adverse drug reactions** |
| --- | --- | --- | --- | --- | --- | --- | --- | --- | --- | --- | --- |
| 1 | Yuan Y, et al. 2022^[1]^ | China | Case | 1 | F | 23 | Bipolar disorder | Ziprasidone | ND | ND | Acne |
| 2 | Walder A, et al. 2009^[2]^ | Switzerland | Case | 1 | F | 55 | Severely depression | Olanzapine | ND | Lithium and pravastatin | Erythematous pigmented skin rash |
| 3 | Torroba Sanz B, et al. 2021^[3]^ | Spain | Case | 1 | M | 60 | Delirium episode | Quetiapine | ND | ND | DRESS Syndrome |
| 4 | Swati C, et al. 2022^[4]^ | India | Case | 1 | M | 26 | Acute Transient Psychotic Disorder | Olanzapine | Within 72 hours | ND | Psoriatic rash |
| 5 | Suvarna P, et al. 2020^[5]^ | India | Case | 1 | M | 18 | Mania | Clozapine | 2 weeks after started on lithium and clozapine | Lithium | SDRIFE |
| 6 | Sundling K, et al. 2019^[6]^ | USA | Case | 1 | M | 64 | Schizoaffective disorder | Paliperidone | Two days after first loading dose | Lamotrigine and venlafaxine | Delayed-Onset Rash |
| 7 | Stammler R, et al. 2022^[7]^ | France | Case | 1 | M | 39 | Persistent hiccups | Chlorpromazine | Twenty days later | ND | Pruritic rash |
| 8 | Andrea Solfanelli, et al. 2013^[8]^ | Italy | Case | 1 | M | 17 | Schizophreniform disorder | Olanzapine | Eight days after the first OLAI administration | ND | Rash |
| 9 | Sogo A, et al. 2022^[9]^ | Japan | Case | 1 | M | 49 | Schizophrenia | Olanzapine | 10 days after olanzapine was resumed | Lansoprazole | Early-onset DIHS/DRESS |
| 10 | Sidhu K, et al. 2010^[10]^ | United States | Case | 1 | M | 26 | ND | Risperidone | After initiation of RLAI treatment. | ND | Diffuse erythematous and maculopapular skin rash |
| 11 | Salgo R, et al. 2011^[11]^ | Germany | Case | 1 | M | 41 | Depression | Zuclopenthixol | ND | ND | Widespread rash |
| 12 | Raz A, et al. 2001^[12]^ | Israel | Case | 1 | M | 34 | Chronic paranoid schizophrenia | Olanzapine | A few days later after take olanzapine | Clonazepam | Hypersensitivity syndrome |
| 13 | Rath A, et al. 2014^[13]^ | India | Case | 1 | M | ND | Psychological symptoms of dementia | Quetiapine | Within 5 days of starting tab. Quetiapine | ND | Exfoliative dermatitis |
| 14 | Rao A, et al. 2012^[14]^ | United States | Cases | 2 | M | Mean age 49 | Schizophrenia | Clozapine | 6 months and 4 months | ND | SDRIFE |
| 15 | Raju K, et al. 2009^[15]^ | NA | Case | 1 | M | ND | Resistant schizophrenia | Clozapine | ND | ND | Eczematous rash |
| 16 | Navarro-Triviño FJ, et al. 2018^[16]^ | Spain | Case | 1 | M | 34 | Paranoid Schizophrenia | Aripiprazole | 6 weeks after beginning treatment with oral Aripiprazole | Mirtazapine | Acneiform rash |
| 17 | Nath S, et al. 2016^[17]^ | India | Case | 1 | M | 21 | Paranoid schizophrenia | Aripiprazole | Nine days after starting aripiprazole | ND | Morbilliform maculopapular skin rashes |
| 18 | Moazez C, et al. 2018^[18]^ | USA | Case | 1 | F | 32 | Schizophrenia, bipolar disorder | Clozapine | ND | ND | DRESS Syndrome |
| 19 | Metin H, et al. 2013^[19]^ | Turkey | Case | 1 | M | 27 | Psychotic symptoms | Amisulpride | Seven days after the initiation of amisulpride | ND | Maculopapular rash |
| 20 | Mallaro H L, et al. 2023^[20]^ | USA | Case | 1 | F | 33 | Acute respiratory distress syndrome | Quetiapine | ND | Lansoprazole | DRESS syndrome |
| 21 | Liu C T, et al. 2011^[21]^ | China | Case | 1 | F | 53 | Chronic schizophrenia | Paliperidone | On the 7 days of hospitalization | ND | Pruritic skin rash |
| 22 | Knight S, et al. 2022^[22]^ | USA | Case | 1 | M | 30 | Paranoia | Olanzapine | 8 weeks after received olanzapine | Vancomycin | DRESS syndrome |
| 23 | Kashani P, et al. 2019^[23]^ | Canada | Case | 1 | M | 7 | Attention Deficit Hyperactivity Disorder | Risperidone | During the third week after initiation of medication | ND | Generalized purpuric, confluent, maculopapular rash |
| 24 | Karmacharya P, et al. 2013^[24]^ | United States | Case | 1 | F | 58 | Bipolar disorder | Olanzapine | 7 days after initiation of olanzapine | ND | Hypersensitivity vasculitis with a diffuse erythematous rash |
| 25 | Kara H, et al. 2018^[25]^ | Turkey | Case | 1 | F | 46 | Depression and obsessive-compulsive disorder | Aripiprazole | 10 days after the onset of aripiprazole | Fluoxetine | A papulopustular acneiform rash |
| 26 | Janardhana P, et al. 2016^[26]^ | India | Case | 1 | M | 20 | Paranoid schizophrenia | Aripiprazole | One week after treatment | ND | Generalized pruritus,generalized scaling of the skin, papules, and ulcers with serous discharge over the dorsum of the hand |
| 27 | Hafeez ZH, et al. 2019^[27]^ | USA | Case | 1 | F | 72 | Schizoaffective disorder | Lurasidone | About 8 days after this increase | ND | An itchy, erythematous, maculopapular rash |
| 28 | Güler HA, et al. 2019^[28]^ | Turkey | Case | 1 | M | 5 | ND | Risperidone | Eighth month of treatment | ND | Diffuse nonpruritic maculopapular skin rash |
| 29 | Gica Ş, et al. 2021^[29]^ | Turkey | Case | 1 | F | 21 | Bipolar affective disorder | Paliperidone  or quetiapine | After she had used 2 doses of the medicine | Paliperidone  and quetiapine | DRESS syndrome |
| 30 | Ghozlane L, et al. 2023^[30]^ | Tunisia | Case | 1 | F | 33 | Bipolar disorder | Chlorpromazine | 24 hours after starting the therapy | ND | DRESS syndrome |
| 31 | Fujimoto S, et al. 2020^[31]^ | Japan | Case | 1 | F | 48 | Schizophrenia | Clozapine | After 2 years of clozapine treatment | ND | AAV skin rash |
| 32 | Fong SY, et al. 2005^[32]^ | China | Case | 1 | F | 55 | Resistant schizophrenia | Clozapine | On day 31 of the medicine | ND | An itchy urticarial rash |
| 33 | Ekinci Ö, et al. 2017^[33]^ | Turkey | Case | 1 | M | 4 | Childhood-onset type conduct  disorder | Risperidone | Three days after the dose increase to 0.5 mg/day twice daily | ND | Bruise-like rash |
| 34 | Duggal MK, et al. 2005^[34]^ | USA | Case | 1 | F | 82 | Delirium | Olanzapine | On the eighth day after initiation of olanzapine | Warfarin | olanzapine |
| 35 | Curtis L, et al. 2020^[35]^ | Ireland | Case | 1 | F | 57 | Schizo-affective disorder | Clozapine | Three months after commencement of clozapine | Dosulepin | Generalised erthyematous pruritic rash |
| 36 | Chu KP J, et al. 2005^[36]^ | Australia | Case | 1 | F | 94 | Delusions and hallucinations | Olanzapine | 4 days after treatment | ND | Generalised erythematous rash |
| 37 | Christen S, et al. 2006^[37]^ | Witzerland | Case | 1 | M | 56 | ND | Olanzapine | 5 days after treatment | Valproate | Acute generalized exanthematous pustulosis |
| 38 | Chae BJ, et al. 2008^[38]^ | Korea | Case | 1 | M | 37 | Bipolar I disorder | Risperidone | On the third day of treatment | Lithium, diazepam, zolpidem and procyclidine hydrochloride | Rash and Desquamation |
| 39 | Bujor CE, et al. 2017^[39]^ | Denmark | Case | 1 | F | 21 | Paranoid schizophrenia | Quetiapine | about 2 months after treatment | Simvastatin | Psoriatic rash |
| 40 | Bryant CA, et al. 2022^[40]^ | USA | Case | 1 | F | 43 | Bipolar disorder | Lurasidone | ND | Lorazepam | DRESS syndrome |
| 41 | Bossed M, et al. 2009^[41]^ | France | Cases | 5 | 3M+2F | 41 | ND | Risperidone | from 3 to 42 days after starting the treatment | ND | Photosensitivity |
| 42 | Bosonnet S, et al. 1997^[42]^ | France | Case | 1 | F | 71 | Schizophrenia | Clozapine | six weeks after treatment | ND | Erythematopustular skin reaction |
| 43 | Biju RA, et al. 2022^[43]^ | India | Case | 1 | M | 78 | ND | Quetiapine | after taking quetiapine for 2 weeks | ND | DRESS syndrome |
| 44 | Ali Guler H, et al. 2018^[44]^ | Turkey | Case | 1 | M | 5 years and 8 months | Aggression, and self-mutilation | Risperidone | ND | No use other medication | Maculopapular skin shedding |
| 45 | Akay BN, et al. 2009^[45]^ | Turkey | Case | 1 | M | 8 | ND | Risperidone | 6 months after starting treatment | ND | Symmetrical drug related intertriginous and flexural exanthema |
| 46 | Affes H, et al. 2020^[46]^ | Tunisia | Case | 1 | F | 37 | Resistant schizophrenia | Chlorpromazine | Oct-18 | Chlorpromazine | Systemic lupus erythematosus |
| 47 | Lihong Zhu, et al. 2016^[47]^ | China | Case | 1 | F | 39 | Affective disorder | Quetiapine | 8 days after treatment | Olanzapine | Erythema multiforme drug eruption |
| 48 | Chenyou Zhu, et al. 1992^[48]^ | China | Case | 1 | M | 30 | Residual schizophrenia | Haloperidol | two weeks after treatment | ND | Rash |
| 49 | Chenyou Zhu, et al. 2000^[49]^ | China | Cases | 4 | 4M | Mean age 32.5 | Paranoid schizophrenia | Haloperidol | - | - | Rash |
| 50 | Qiujuan Zhou, et al. 2013^[50]^ | China | Case | 1 | M | 26 | Schizophrenia | Amisulpride | 5 days after treatment | Olanzapine | Rash |
| 51 | Qiujuan Zhou, et al. 2012^[51]^ | China | Case | 1 | M | 25 | Paranoid schizophrenia | Olanzapine | 13 days after treatment | - | Measles-like rash |
| 52 | Guangyan Zhou, et al. 2010^[52]^ | China | Case | 1 | M | 27 | Schizophrenia | Aripiprazole | the day after treatment | Sulpiride | Psoriasis |
| 53 | Guangyan Zhou, et al. 2003^[53]^ | China | Case | 1 | M | 50 | Catatonic syndrome | Sulpiride | 4 days after treatment | ND | Bullous eruption |
| 54 | Haizhen Zhong, et al. 2014^[54]^ | China | Case | 1 | F | 40 | Schizophrenia | Perospirone | 3 days after treatment | ND | Red aculopapular rash |
| 55 | Min Zhao, et al. 2007^[55]^ | China | Case | 1 | F | 28 | Affective disorder | Chlorpromazine | 4 days after treatment | ND | Exfoliative dermatitis |
| 56 | Zhenfang Zhang, et al. 1998^[56]^ | China | Case | 1 | M | 26 | Schizophrenia | Clozapine | About 4 months after this increase | ND | Symmetric acute eczema-like changes in both lower limbs |
| 57 | Xiaomin Zhang, et al. 1990^[57]^ | China | Case | 1 | F | 32 | Schizophrenia | Clozapine | 2 months after treatment | ND | Lichenoid rash |
| 58 | Li Zhang, et al. 2007^[58]^ | China | Case | 1 | M | 31 | Undefined schizophrenia | Sulpiride | 15 days after treatment | ND | Exfoliative dermatitis |
| 59 | Fengxian Zhang, et al. 2002^[59]^ | China | Case | 1 | M | 33 | Schizophrenia | Risperidone | 20 days after treatment | ND | Red rash |
| 60 | Mei Yuan, et al. 2015^[60]^ | China | Case | 1 | F | 45 | ND | Risperidone | 3 days after treatment | ND | Erythema |
| 61 | Dongshan Yu, et al. 2011^[61]^ | China | Cases | 3 | 3M | Mean age 22.7 | - | Clozapine | - | - | Pruritus in heat |
| 62 | Yinzhen Ye, et al. 1996^[62]^ | China | Case | 1 | M | 24 | Schizophrenia | Chlorpromazine | 9 days after treatment | ND | Sporadic rash |
| 63 | Jiaqing Yang, et al. 2002^[63]^ | China | Case | 1 | M | 45 | Schizophrenia | Clozapine | 6 days after treatment | ND | Epidermolysis bullosa |
| 64 | Huiyun Yang, et al. 2002^[64]^ | China | Case | 1 | M | 25 | Schizophrenia | Sulpiride | 18 days after treatment | ND | Exfoliative dermatitis |
| 65 | Guowei Xue, et al. 1982^[65]^ | China | Cases | 3 | 3F | Mean age 27.7 | Schizophrenia | Clozapine | - | ND | 2 cases of rash |
| 66 | Xi Zhang, et al. 2002^[66]^ | China | Case | 1 | F | 40 | Schizophrenia | Clozapine | ND | ND | Allergic dermatitis |
| 67 | Yatao Wu, et al. 1997^[67]^ | China | Case | 1 | F | 23 | Schizophreniform psychosis | Chlorpromazine | the second day after treatment | ND | Papule |
| 68 | Xiumei Wu, et al. 2005^[68]^ | China | Case | 1 | F | 32 | Schizophreniform psychosis | Perphenazine | 8 days after treatment | ND | Exfoliative dermatitis |
| 69 | Guihong Wu, et al. 2014^[69]^ | China | Cases | 4 | 3M+1F | Mean age 30.2 | 3 cases of schizophrenia,mania | Aripiprazole | 7-10 days after treatment | ND | Red rash |
| 70 | Yingge Wu, et al. 2006^[70]^ | China | Case | 1 | M | 28 | Schizophrenia | Aripiprazole | 2 days after treatment | ND | Papules |
| 71 | Yuguan Wen, et al. 2000^[71]^ | China | Case | 1 | F | 34 | Schizophrenia | Clozapine | 9 days after treatment | ND | Urticaria |
| 72 | Yingchan Wang, et al. 2011^[72]^ | China | Case | 1 | F | 25 | Schizophrenia | Quetiapine | 7 days after treatment | ND | Erythema multiforme drug eruption |
| 73 | Yizhen Wang, et al. 2001^[73]^ | China | Case | 1 | F | 19 | Schizophrenia | Clozapine | 20min after treatment | ND | Epidermolysis bullosa |
| 74 | Xiaoli Wang, et al. 1996^[74]^ | China | Cases | 2 | 2F | Mean age 35.5 | Schizophrenia | Haloperidol | 7 days and 21h after treatment | ND | Drug-induced vesicular eruption |
| 75 | Weihang Wang, et al. 2009^[75]^ | China | Case | 1 | F | 19 | Schizophrenia | Aripiprazole | 5 days after treatment | ND | Exfoliative dermatitis |
| 76 | Shiming Wang, et al. 1999^[76]^ | China | Case | 1 | F | 35 | Schizophrenia | Risperidone | 74 days after treatment | ND | Urticaria |
| 77 | Jinyu Wang, et al. 2001^[77]^ | China | Case | 1 | F | 70 | Schizophrenia | Risperidone | 3 days after treatment | ND | Drug-induced vesicular eruption |
| 78 | Jian Wang, et al. 1985^[78]^ | China | Case | 1 | F | 31 | ND | Chlorpromazine | a few minutes later | ND | rubella like rash |
| 79 | Huiying Wang, et al. 1980^[79]^ | China | Case | 1 | F | 75 | ND | Chlorpromazine | ND | ND | Lichenoid rash |
| 80 | Heqiu Wang, et al. 2004^[80]^ | China | Case | 1 | M | 29 | Schizophrenia | Quetiapine | About 7 days after this increase | ND | Rash |
| 81 | Airong Wang, et al. 1996^[81]^ | China | Case | 1 | M | 24 | Schizophrenia | Sulpiride | 6 days after treatment | ND | Vesicular eruption |
| 82 | Zhiqiu Tang, et al. 1991^[82]^ | China | Cases | 3 | 3M | Mean age 22.3 | Schizophrenia | Haloperidol | 30 days, 3days and 6days after treatment | ND | Dermatitis medicamentosa |
| 83 | Zhijian Tan, et al.1989^[83]^ | China | Case | 1 | F | 19 | Schizophrenia | Chlorpromazine | 16 days after treatment | ND | Toxic epidermal necrolysis type drug eruption |
| 84 | Jiaxu Qin, et al. 1993^[84]^ | China | Case | 1 | M | 20 | Schizophrenia | Chlorpromazine | About 7 days after this increase | ND | Exfoliative dermatitis |
| 85 | Zhenxiao Sun, et al. 2013^[85]^ | China | Case | 1 | F | 56 | Somatoform disorders | Olanzapine | 2 days after treatment | Duloxetine | Erythema multiforme drug eruption |
| 86 | Xiaoxia Sun, et al. 2008^[86]^ | China | Case | 1 | M | 11 | Tourette syndrome | Haloperidol | 7 days after treatment | ND | Thrombocytopenic purpura |
| 87 | Chuanreng Sun, et al. 1999^[87]^ | China | Case | 1 | M | 21 | Affective disorder | Chlorpromazine | 4 days after treatment | ND | Exfoliative dermatitis |
| 88 | Lianghua Shi, et al. 1999^[88]^ | China | Case | 1 | M | 26 | Schizophreniform psychosis | Clozapine | 3 days after retreatment | ND | Erythema multiforme |
| 89 | Jun Shen, et al. 1989^[89]^ | China | Case | 1 | M | 21 | Schizophrenia | Clozapine | 10 days after treatment | ND | Epidermolysis bullosa |
| 90 | Yan Peng, et al. 2020^[90]^ | China | Case | 1 | M | 30 | Schizophrenia | Clozapine | 11 days after treatment | ND | Epidermolysis bullosa |
| 91 | Lihong Pan, et al. 2016^[91]^ | China | Case | 1 | F | 30 | Schizophrenia | Olanzapine | ND | ND | Pruritus and paresthesia |
| 92 | Hongying Mi, et al. 1999^[92]^ | China | Case | 1 | M | 10 months | Calm | Chlorpromazine | 10min after treatment | ND | Red rash |
| 93 | Aijun Lu, et al. 2014^[93]^ | China | Case | 1 | M | 47 | Schizophrenia | Clozapine | 7 days after treatment | ND | Papules |
| 94 | Yongping Liu, et al. 2013^[94]^ | China | Case | 1 | F | 42 | Schizophrenia | Risperidone | ND | ND | Allergic dermatitis |
| 95 | Qingyun Liu, et al. 1996^[95]^ | China | Cases | 2 | 2F | Mean age 27.5 | Paranoia schizophrenia, schizophreniform psychosis | Haloperidol | The second day,21 days after treatment | ND | Dermatitis |
| 96 | Linlin Liu, et al. 2011^[96]^ | China | Case | 1 | M | 31 | Undifferentiated schizophrenia | Quetiapine | 2 months after treatment | ND | Urticaria |
| 97 | Junlin Liu, et al. 2003^[97]^ | China | Case | 1 | M | 30 | Schizophrenia | Risperidone | 3 months after treatment | ND | Morbilliform erythema |
| 98 | Jingnian Liu, et al. 2003^[98]^ | China | Cases | 2 | M | 35 | Schizophrenia | Clozapine | 8 days, 6days when treatment | ND | Epidermolysis bullosa |
| 99 | Hengwei Liu, et al. 1997^[99]^ | China | Case | 1 | M | 20 | Drug-related acute poisonings | Clozapine | about 5h after treatment | ND | Erythema multiforme drug eruption |
| 100 | Haiyan Liu, et al. 2001^[100]^ | China | Case | 1 | F | 26 | Psychiatric history | Sulpiride | 2 days after treatment | ND | Epidermolysis bullosa |
| 101 | Aiqin Liu, et al. 2008^[101]^ | China | Case | 1 | M | 24 | Schizophrenia | Risperidone | The second day after treatment | ND | Red maculopapule |
| 102 | Zhuxin Lin, et al. 1995^[102]^ | China | Case | 1 | F | 38 | Schizophrenia | Clozapine | The eighth day after treatment | ND | Red papule |
| 103 | Yunquan Li, et al. 1994^[103]^ | China | Case | 1 | F | 52 | Schizophrenia | Clozapine | 7 days after treatment | ND | Rash |
| 104 | Xiaoxu Li, et al. 2002^[104]^ | China | Case | 1 | F | 28 | Schizophreniform psychosis | Perphenazine | 9 days after treatment | ND | Exfoliative dermatitis |
| 105 | Wenxia Li, et al. 1989^[105]^ | China | Case | 1 | M | 18 | Schizophrenia | Clozapine | 19 days after treatment | ND | Maculae |
| 106 | Shiping Li, et al. 1995^[106]^ | China | Case | 1 | F | 20 | Affective disorder | Clozapine | 20 days after treatment | ND | Rash |
| 107 | Qingfang Li, et al. 1996^[107]^ | China | Case | 1 | F | 30 | Schizophrenia | Sulpiride | 14 days after treatment | ND | Rash |
| 108 | Ping Li, et al. 2001^[108]^ | China | Case | 1 | F | 16 | Schizophrenia | Sulpiride | 4 days after treatment | ND | Erythema multiforme drug eruption |
| 109 | Ling Li, et al. 1997^[109]^ | China | Case | 1 | M | 60 | Psychogenic mental disorders | Perphenazine | About 2 days after this increase | ND | Vesicular eruption |
| 110 | Jianfen Li, et al. 2005^[110]^ | China | Cases | 2 | M+F | Mean age 56 | Schizophrenia | Chlorpromazine | 3weeks, 6weeks after treatment | ND | Exfoliative dermatitis |
| 111 | Yanyan Jiang, et al. 2009^[111]^ | China | Case | 1 | F | 30 | Schizophrenia | Risperidone | 5 days after treatment | ND | Red aculopapular rash |
| 112 | Xiaohong Hu, et al. 2013^[112]^ | China | Case | 1 | M | 18 | Schizophrenia | Sulpiride | 4 days after treatment | Risperidone and cefalexin | Rash |
| 113 | Manji Hu, et al. 2012^[113]^ | China | Case | 1 | M | 22 | Schizophrenia | Amisulpride | 6 days after treatment | ND | Urticaria |
| 114 | Fusheng Hu, et al. 2000^[114]^ | China | Case | 1 | M | 36 | Schizophrenia | Risperidone | 2 years and 3months after treatment | ND | Papuloid skin damage |
| 115 | Jiakun Hong, et al. 2022^[115]^ | China | Case | 1 | M | 25 | Schizophrenia | Ziprasidone | 72 days after treatment | ND | Dermatitis |
| 116 | Guoqi He, et al. 2008^[116]^ | China | Cases | 3 | 3F | Mean age 36 | Hysterical psychosis, schizophrenia, mania | Quetiapine | 1-2 weeks after treatment | ND | Allergic dermatitis |
| 117 | Zhengmei Guo, et al. 2016^[117]^ | China | Case | 1 | F | 20 | Mania | Quetiapine | 5 days after treatment | ND | Allergic dermatitis |
| 118 | Yuhua Guo, et al. 2001^[118]^ | China | Case | 1 | M | 24 | Schizophrenia | Risperidone | about 1.5 years after treatment | ND | Rash |
| 119 | Yingli Guo, et al. 2016^[119]^ | China | Cases | 3 | 3F | Mean age 39.7 | stuporous state | Sulpiride | 13 days,22 days and 3days after treatment | ND | Epidermolysis bullosa |
| 120 | Weiqiang Gu, et al. 2008^[120]^ | China | Case | 1 | F | 42 | Schizophrenia | Chlorpromazine | 9 days after treatment | ND | Exfoliative dermatitis |
| 121 | Weiqiang Gu, et al. 2009^[121]^ | China | Case | 1 | M | 50 | Schizophrenia | Clozapine | 33 days after treatment | ND | Rash |
| 122 | Mingzhi Gu, et al. 1994^[122]^ | China | Case | 1 | F | 37 | Mental disorders after craniocerebral injury | Sulpiride | 2 days after treatment | ND | Whole-body rose papules |
| 123 | Bingzhu Gao, et al. 1997^[123]^ | China | Case | 1 | M | 26 | Schizophrenia | Clozapine | 4 days after treatment | ND | Bullous eruption |
| 124 | Zhiping Fang, et al. 2008^[124]^ | China | Case | 1 | M | 32 | Schizophrenia | Risperidone | 20 days after treatment | ND | Dermatitis |
| 125 | Xiaoting Fang, et al. 2016^[125]^ | China | Case | 1 | F | 60 | Bipolar disorder | Olanzapine | 4 days after treatment | ND | Erythema multiforme drug eruption |
| 126 | Ke Du, et al. 2002^[126]^ | China | Case | 1 | F | 10 | Tourette syndrome | Tiapride | 4 days after treatment | ND | Rash |
| 127 | Yi Dong, et al. 1996^[127]^ | China | Case | 1 | M | 23 | ND | Clozapine | 54 days after treatment | Carbamazepine | Exfoliative dermatitis |
| 128 | Jing Deng, et al. 2001^[128]^ | China | Case | 1 | F | 40 | Schizophrenia | Clozapine | 3 days after treatment | ND | Urticaria |
| 129 | Wenjun Shan, et al. 2002^[129]^ | China | Case | 1 | M | 35 | Wart | Chlorpromazine | 2 days after treatment | ND | Papule |
| 130 | Licheng Chen, et al. 2003^[130]^ | China | Case | 1 | F | 13 | Childhood mental disorders | Risperidone | 2h after treatment | Perphenazine | Miliary rash |
| 131 | Dayong Chen, et al. 1982^[131]^ | China | Case | 1 | F | 69 | Atopic dermatitis | Chlorpromazine | 3 days after treatment | ND | Erythema multiforme drug eruption |
| 132 | Xiuying Cao, et al. 2009^[132]^ | China | Case | 1 | F | 20 | ND | Clozapine | 10 days after treatment | ND | Exfoliative dermatitis |
| 133 | Hengbing Cao, et al. 2013^[133]^ | China | Case | 1 | F | 42 | Paranoid schizophrenia | Risperidone | 48 days after treatment | Alprazolam | Whole-body rash |
| 134 | Zhengrong Ang, et al. 2001^[134]^ | China | Case | 1 | F | 46 | Schizophrenia | Clozapine | 4 days after treatment | ND | Rash |
| 135 | Zhengrong Ang, et al. 2008^[135]^ | China | Case | 1 | M | 55 | Schizophreniform psychosis | Aripiprazole | 1 months after treatment | ND | Rash |
| 136 | Jiling Wang, et al. 1988^[136]^ | China | Case | 1 | M | 35 | Schizophrenia | Clozapine | 5 days after treatment | ND | Rash |
| 137 | Ruiming Zhu, et al. 1996^[137]^ | China | Cases | 3 | M+2F | Mean age 20 | Mania | 2 case of clozapine treatment, hlorpromazine | 2 days,7 days and 5 days after treatment | — | Rash |
| 138 | Chunyang Li, et al. 2001^[138]^ | China | Case | 1 | F | 44 | Schizophrenia | Sulpiride and clozapine | 2 days after treatment | Chlorpromazine | Rash |
| 139 | Song He, et al. 2002^[139]^ | China | Cases | 7 | 2M+5F | ND | 5 cases of schizophrenia,1 case of bipolar disorder and 1 case of Reactive psychosis | Chlorpromazine | 14-42 days after treatment | ND | Exfoliative dermatitis |

ND: information not described.

**References:**

[1] Yuan Y, Li X, Jiang X, Li Z, Ou Y, Li Z. Acne caused by ziprasidone in a young patient with bipolar disorder: A case report. Front Psychiatry. 2022; 13:948977. https://doi.org/10.3389/fpsyt.2022.948977.

[2] Walder A, Baumann P. Mood stabilizer therapy and pravastatin: higher risk for adverse skin reactions?. Acta Medica (Hradec Kralove). 2009; 52(1):15-8. https://doi.org/10.14712/18059694.2016.101.

[3] Torroba Sanz B, Mendez Martínez E, Cacho Asenjo E, Aquerreta Gonzalez I. Permanent renal sequelae secondary to drug reaction with eosinophilia and systemic symptoms (DRESS) syndrome induced by quetiapine. Eur J Hosp Pharm. 2021; 28(5):285-288. https://doi.org/10.1136/ejhpharm-2019-002149.

[4] Swati C, Singh H, Bunty S. Case report of olanzapine induced psoriasis in a young adult male with Acute Transient Psychotic Disorder. Indian J Psychiatry. 2022; 64(SUPPL 3): S613-S614.

[5] Suvarna P, Kayarkatte MN, Shenoi SD, Jaiprakash P A rare case of clozapine-induced symmetrical drug-related intertriginous and flexural exanthema with vasculitis-like lesions. Contact Dermatitis. 2020; 82(5):318-320. https://doi.org/10.1111/cod.13468.

[6] Sundling K, Held J, Narang P, Lippmann S. Paliperidone Injection-Associated Delayed-Onset Rash. Prim Care Companion CNS Disord.2019; 21(1):18l02323. https://doi.org/10.4088/PCC.18l02323.

[7] Stammler R, Ackermann F, Vasse M, Verrat A, David A, Horodyckid C, Gratieux J, Marroun I, Groh M, Roumier M, Paule R. Life-threatening chlorpromazine-induced acquired haemophilia A in a patient with a cavernous malformation involving the medulla oblongata. Rev Med Interne. 2022; 43(12):739-742. https://doi.org/10.1016/j.revmed.2022.08.009.

[8] Solfanelli A, Curto M, Dimitri-Valente G, Kotzalidis GD, Gasperoni C, Sani G, Manfredi G, Rapinesi C, Comparelli A, Girardi P. Skin rash occurring with olanzapine pamoate, but not with oral olanzapine, in a male with juvenile idiopathic arthritis. J Child Adolesc Psychopharmacol. 2013; 23(3):232-4. https://doi.org/10.1089/cap.2012.0121.

[9] Sogo A, Horiuchi H, Ueda T, Miyazaki H, Furuya R. Early-Onset Drug Hypersensitivity Syndrome in a Man With Pneumonia Due to Pre-sensitization to Olanzapine. Cureus. 2022; 14(6): e26374. https://doi.org/10.7759/cureus.26374.

[10] K. Sidhu, H. Saggu, L. Lachover, J.T. Dziuba, Rare case report of rash associated with risperidone long-acting injection. Primary Psychiatry. 2010; 17(8):38-40.

[11] Salgo R, Boehncke WH. A 41 year old man with an itchy rash. BMJ. 2011; 343: d7320. https://doi.org/10.1136/bmj.d7320.

[12] Raz A, Bergman R, Eilam O, Yungerman T, Hayek T. A case report of olanzapine-induced hypersensitivity syndrome. Am J Med Sci. 2021; 321(2):156-158. http://dx.doi.org/10.1097/00000441-200102000-00008.

[13] A. Rath, S. Panse, I.S. Netto, A.V. Pawar. Quetiapine-induced exfoliative dermatitis: A rare complication. Indian J Psychiatry. 2014; 56: S64.

[14] Rao A, Francis N, Morar N. Clozapine-induced symmetrical drug-related intertriginous and flexural exanthema: first reported cases. Br J Dermatol. 2021; 166(5):1142-1143. http://dx.doi.org/10.1111/j.1365-2133.2011.10758.x.

[15] K. Raju, R. Singh, J. Southern, J. Darroch. Clozapine induced rash: Case report of successful desensitisation. J Neurol Neurosurg Psychiatry. 2009; 80(7): 827.

[16] Navarro-Triviño FJ, de Jaime Ruiz P, Porras Segovia A, Garrido Torres-Puchol V. Oral Isotretinoin for the treatment of Aripiprazol-induced acneiform rash. Dermatol Ther. 2018; 31(4):e12637. http://dx.doi.org/10.1111/dth.12637.

[17] Nath S, Rehman S, Kalita KN, Baruah A. Aripiprazole-induced skin rash. Ind Psychiatry J. 2016; 25(2):225-227. http://dx.doi.org/10.4103/0972-6748.207862.

[18] Moazez C, Rudha Y, Narang N, Younger T. A Rare Presentation of Clozapine-Induced DRESS Syndrome. Case Rep Med. 2018:1346351. http://dx.doi.org/10.1155/2018/1346351.

[19] H. Metin, U. Ozer, S. Ozen, G. Ozgen. Amisulpride-induced maculopapular rash: a case report. Bulletin of Clinical Psychopharmacology. 2013; 23: S151-S152.

[20] Mallaro HL, Rosenthal LJ. DRESS syndrome: quetiapine associated case report and literature review. Int Clin Psychopharmacol. 2023; 38(5):356-360. http://dx.doi.org/10.1097/YIC.0000000000000474.

[21] Liu CT, Hsieh TH. Osmotic-controlled release oral delivery system (OROS) paliperidone-related pruritic rash. J Clin Psychopharmacol. 2011; 31(4):525-6. http://dx.doi.org/10.1097/JCP.0b013e318222b958.

[22] S. Knight, A. Hardeman, L. Wild. Paranoid about rashes: olanzapine induced drug reaction with eosinophilia and systemic symptoms. Ann Allergy Asthma Immunol. 2022; 129(5): S93.

[23] P. Kashani, S. Afraz, S. Lavi. Serum sickness-like reaction to a second generation antipsychotic drug. LymphoSign Journal. 2019; 6(4):136-140.

[24] P. Karmacharya, M.R. Aryal, A.A. Donato. Olanzapine induced cutaneous leukocytoclastic vasculitis. J Gen Intern Med. 2013; 28: S366.

[25] H. Kara, Ö. Özçelik, M.M. Balci, M.M. Kuloǧlu, T. Tomar. Aripiprazole-induced allergy: Case report. Psychiatry and Clinical Psychopharmacology. 2018; 28:140-141.

[26] Janardhana P, Nagaraj AK, Basavanna PL. Risperidone-induced skin rash. Indian J Psychiatry. 2016; 58(1):106-107. http://dx.doi.org/ 10.4103/0019-5545.174407.

[27] Hafeez ZH. Exanthematous Rash With Lurasidone in an Elderly Woman With Schizoaffective Disorder. Prim Care Companion CNS Disord. 2019; 21(2):18l02370. http://dx.doi.org/ 10.4088/PCC.18l02370.

[28] H.A. Güler, S. Türkoğlu, D. Güler. Maculopapular rash associated with risperidone in a child. Psychiatry and Clinical Psychopharmacology. 2019; 29(4):527-528.

[29] Gica Ş, Elmaci ZC, Balaban ÖD, Karamustafalioglu N. Antipsychotic-Induced Drug Rash With Eosinophilia and Systemic Symptoms Syndrome: A Case Report. Am J Ther. 2021; 28(2):e253-e254. http://dx.doi.org/10.1097/MJT.0000000000001062.

[30] Ghozlane L, Asma J, Ahmed Z, Ons C, Sarrah K, Riadh D, Sihem EA. Antipsychotics Induced Drug Reaction with Eosinophilia and Systemic Symptoms (DRESS) Syndrome: Literature Review and a Report of a Suspected Case Related to Chlorpromazine. Curr Drug Saf. 2023; 18(4):571-575. http://dx.doi.org/10.2174/1574886317666220603104837.

[31] Fujimoto S, Ueda N, Nishimura N, Naito A, Hiura J, Mashiba K, Ikai A, Marutsuka K, Mizuno K. Clozapine-induced antineutrophil cytoplasmic antibody-associated vasculitis: a case report. Mod Rheumatol Case Rep. 2020; 4(1):70-73. http://dx.doi.org/ 10.1080/24725625.2019.1628413.

[32] Fong SY, Au Yeung KL, Tosh JM, Wing YK. Clozapine-induced toxic hepatitis with skin rash. J Psychopharmacol. 2005; 19(1):107. http://dx.doi.org/10.1177/0269881105047287.

[33] Ö. Ekinci, M.E. Tan, M. Kalinli. Risperidone-Induced Bruise-Like Rash in a Child. Psychiatry and Clinical Psychopharmacology.2017; 27:128.

[34] Duggal MK, Singh A, Arunabh, Lolis JD, Guzik HJ. Olanzapine-induced vasculitis. Am J Geriatr Pharmacother. 2005; 3(1):21-24. http://dx.doi.org/10.1016/j.amjopharm.2005.03.003.

[35] Curtis L, Hallahan B, Byrne F. A clozapine-induced hypersensitivity reaction. Ir J Psychol Med. 2020; 37(2):122-125. http://dx.doi.org/ 10.1017/ipm.2017.45.

[36] K.P.J. Chu, K. Nyfort-Hansen. Hypothermia associated with olanzapine, J Pharm Pract Res. 2005; 35(3) : 216-218.

[37] Christen S, Gueissaz F, Anex R, Zullino DF. Acute generalized exanthematous pustulosis induced by olanzapine. Acta Medica (Hradec Kralove). 2006; 49(1):75-76.

[38] Chae BJ, Kang BJ. Rash and desquamation associated with risperidone oral solution. Prim Care Companion J Clin Psychiatry. 2008; 10(5):414-5. http://dx.doi.org/10.4088/pcc.v10n0511e.

[39] Bujor CE, Vang T, Nielsen J, Schjerning O. Antipsychotic-associated psoriatic rash - a case report. BMC Psychiatry. 2017; 17(1):242. http://dx.doi.org/10.1186/s12888-017-1411-2.

[40] Bryant CA, Cinibulk M, Manchee C, Cho SH. Running Out of Options: A Case of DRESS with Possible Neosensitization to Lurasidone in an Acutely Manic, Psychotic, and Catatonic Patient. J Acad Consult Liaison Psychiatry. 2022; 63: S180-S181. http://dx.doi.org/10.1016/j.jaclp.2022.10.140.

[41] M. Bossed, A. Molia, E. Herlem, F. Roca, M.L. Germain, T. Trenque. Risperidone-induced photosensitivity. Drug safety. 2009; 32(10) : 898.

[42] Bosonnet S, Dandurand M, Moati L, Guillot B. Acute generalized exanthematic pustulosis after intake of clozapine (leponex). First case. Ann Dermatol Venereol. 1997; 124(8):547-8. PMID: 9740850.

[43] R.A. Biju, S. Davis, G. Sanal. Quetiapine-induced drug rash with eosinophilia and systemic symptom syndrome. Journal of Medical Sciences. 2022; 42(5): 242-244. http://dx.doi.org/10.4103/jmedsci.jmedsci_82_21.

[44] H. Ali Guler, A. Kandeger, D. Guler, S. Turkoglu. Risperidone-induced maculopapular rash in a paediatric patient. Psychiatry and Clinical Psychopharmacology. 2018; 28: 144-145. http://dx.doi.org/10.1080/24750573.2018.1467600.

[45] Akay BN, Sanli H. Symmetrical drug-related intertriginous and flexural exanthem due to oral risperidone. Pediatr Dermatol. 2009; 26(2):214-6. http://dx.doi.org/10.1111/j.1525-1470.2009.00882.x.

[46] H. Affes, I. Chaari, I. Feki, S. Hammami, J. Masmoudi, K. Zeghal, K. Ksouda. Phenothiazine-induced systemic lupus erythematosus: A case report. European Psychiatry. 2020; 63:S491-S492.

[47] Lihong Zhu, Xiaochun Wang. Nursing experience of a case of bipolar disorder hypomania with severe erythema multiforme drug eruption. J China Health Care & Nutrition.2016; 26(7):375.

[48] Chengyou Zhu, Hongxu Wang, Yuedai Pang. A case report of fever and rash caused by haloperidol. Chin J Nervous Mental Dis 1992; (2):124.

[49] Chengyou Zhu, Dianbao Ren. Four cases of haloperidol sensitizative. Occupation and Health. 2000; 16(6):40.

[50] Qiujuan Zhou, Xiaoyan Wu, Falin Qu. A case report of drug eruption caused by amisulpride tablets. Chin J Pharmacov. 2013; 10(09): 575-576.

[51] Qiujuan Zhou, Xiaoyan Wu, Falin Qu. A case report of morbilliform drug eruption caused by olanzapine. Chin J Pharmacov 2012; 9(10):639-640.

[52] Guangyan Zhou. A case report of psoriasis caused by aripiprazole. J Clin Psychiatry. 2010; 20(5): 314.

[53] Guangyan Zhou. A case report of bullous drug eruption caused by sulpiride. Shangdong Arch Psychiatry. 2003; 16(4):228.

[54] Haizhen Zhong, Hao Jiang, Jing Wang. A case report of skin rash caused by piropilone. Journal of Psychiatry. 2007; 27(4): 287. http://dx.doi.org/3969/j.issn.1009-7201.2014.04.016.

[55] Min Zhao. Exfoliative dermatitis drug eruption caused by chlorpromazine: a case report. Journal of Psychiatry. 2007; 20(5):314.

[56] Zhenfang Zhang, Guiling Wang. A case report of symmetrical acute eczema-like changes of both lower limbs caused by clozapine. Modern Journal of Integrated Chinese and Western Medicine.1998; 7(2):242-243.

[57] Xiaomin Zhang, Shiyin Li, Li Shen, Xueyi Jiang. A case report of lichenoid drug eruption. J Beijing Med Univ. 1990; 22(1):70.

[58] Li Zhang, Jian Feng. Nursing care of a patient with exfoliative dermatitis eruption induced by sulpiride. Chinese Nursing Research. 2007; 21(19):1781-1782.

[59] Fengxian Zhang. A case report of drug rash caused by risperdal. Journal of Zhongyuan Psychiatric medicine. 2002; 8(2):70.

[60] Meiyuan. A case report of drug hypersensitivity syndrome. J Clin Psychiatry. 2015; 25(3):184-184.

[61] Dongshan Yu. Three cases of febrile itching attack caused by clozapine. Sichuan Mental Health. 2011; 24(03):141.

[62] Yinzhen Ye, Jian Wang. A case report of multisystem reaction caused by antipsychotic drugs. Mil Med J S Chin. 1996; (2):170.

[63] Jiaqing Yang. A case report of epidermolysis bullosa caused by clozapine. Chin J Nervous Mental Dis. 2002; 28(3):165.

[64] Huiyun Yang. Exfoliative dermatitis caused by sulpiride: a case report. Chinese Journal of Psychiatry. 2002; 35(1):20.

[65] Guowei Xue. A case report of seizures and rash caused by domestic clozapine. Zhongjiqikan. 1982; (8) 60-61.

[66] Xi Zhang, Tiemei Zhao, Xiumin Guo. Allergic dermatitis induced by high-dose clozapine. Clinical Misdiagnosis AND Mistherapy. 2002; 15(1) :68.

[67] Yatao Wu. A case report of One patient was allergic to chlorpromazine clozapine. Journal of Zhongyuan Psychiatric medicine. 1997; 3(3) :169.

[68] Xiumei Wu, Xinmei Liu, Dongchun Yue. Nursing of a psychotic patient with exfoliative dermatitis caused by perphenazine. Nanfang Journal of Nursing. 2005; 12(6):91. http://dx.doi.org/10.3969/j.issn.1008-9969.2005.06.044.

[69] Guihong Wu. Four cases of skin allergic reaction caused by aripiprazole. Chinese Journal of Pharmacoepidemiology. 2014; 23(6):396-397. http://dx.doi.org/10.19960/j.cnki.issn1005-0698.2014.06.023.

[70] Yingge Wu, Lin Yu, Li Yang. A case report of urticaria caused by aripiprazole. Journal of Clinical Psychosomatic Diseases. 2006; 12(5): Ⅴ. http://dx.doi.org/10.3969/j.issn.1672-187X.2006.05.048.

[71] Yuguan Weng, Haoying Hu, Jianxiong Guo. A case report of urticaria caused by clozapine. Chinese Journal of Clinical Pharmacy. 2000; 9(3): 182. http://dx.doi.org/10.3969/j.issn.1007-4406.2000.03.028.

[72] Yingchan Wang. A case report of quetiapine-induced erythema multiforme. Shanghai Archives of Psychiatry. 2011; 23(4):245-246.

[73] Yizhen Wang. Epidermolysis bullosa induced by clozapine: a case report. Zhongguo Xiangcun Yiyao. 2001; (5):39.

[74] Xiaoli Wang. Two cases of haloperidol induced vesicular drug eruption. Psychiatry in Shandong. 1996; 9(4):19.

[75] Weihang Wang, Wanqiu Na, Qiuxia Xu. Exfoliative dermatitis caused by aripiprazole: a case report. Chinese Journal of Psychiatry. 2009; 42(3):157. http://dx.doi.org/10.3760/cma.j.issn.1006-7884.2009.03.011.

[76] Shiming Wang, Yaqin Chen, Hongfa Zhang. A case report of giant urticaria caused by risperidone. Chin J Nervous Mental Dis. 1999; 25(4): 253. http://dx.doi.org/10.3969/j.issn.1002-0152.1999.04.050.

[77] Jinyu Wang. A case report of vesicular drug eruptions caused by Risperdal. Shandong Archives of Psychiatry. 2001; 14(3):169. http://dx.doi.org/10.3969/j.issn.1009-7201.2001.03.028.

[78] Jian Wang, Hong Zhu. A case report of acute interstitial porphyria complicated with short stature and chlorpromazine allergy. Tianjin Med J.1985; (9):543.

[79] Huiying Wang. Lichenoid dermatitis caused by chlorpromazine phototoxicity. Foreign Medical Sciences. 1980; (4):244-245.

[80] Heqiu Wang, Suzhen Wang. High dose quetiapine caused rash in 1 case. Psychiatry in Shanghai. 2004; 16(1):36-37.

[81] Airong Wang, Peisheng Wang, Hongxia Liu, Yutang Jia. A case report of blister rash caused by intravenous sulpiride. Chin J Nervous Mental Dis. 1996; (2):77.

[82] Zhiqiu Tang. Three cases of drug-induced dermatitis caused by haloperidol. New Drugs Clin Rem. 1991; 10 (6):339-340.

[83] Zhijian Tan. A case report of toxic epidermal necrolysis drug eruption caused by chlorpromazine. Acta Univ Med Tongji. 1989; (3):155.

[84] Jiaxu Qin. Systemic exfoliative dermatitis induced by chlorpromazine: a case report. Youjiang Medical Journal. 1993; (1):22-23.

[85] Zhenxiao Sun, Xiangfen Yu. A case report of drug eruption of facial erythema multiforme caused by oral olanzapine. Chin J New Drugs Clin Rem. 2013; 32(12):1001-1002.

[86] Xiaoxia Sun, Baoguo Shao. A case report of haloperidol induced thrombocytopenic purpura. Qinghai Medical Journal. 2008; 38(7):77.

[87] Chuanreng Sun. Exfoliative dermatitis drug eruption caused by chlorpromazine: a case report. Chinese Journal of Psychiatry.1999; (4): 22.

[88] Lianghua Shi. Care of erythema multiforme caused by clozapine. Journal of Zhenjiang Medical College. 1999; (4):169. http://dx.doi.org/10.13312/j.issn.1671-7783.1999.04.179.

[89] Jun Shen, Ming Zhai. Epidermolysis bullosa with brachial plexus injury caused by clozapine: a case report. Chin J Nervous Mental Dis. 1989; (1):31.

[90] Yan Peng, Junpeng Li. A case report of epidermolysis bullosa caused by mistakenly taking large dose of clozapine. Jilin Medical Journal. 2020; 41(3): 762-763.

[91] Lihong Pan. A case report of pruritus and paresthesia caused by olanzapine. Journal of Clinical Psychiatry. 2016; 26(6):387-387.

[92] Hongying Mi, Ping Li. A case report of anaphylactic shock in child caused by chlorpromazine. Chinese Journal of Pharmacoepidemiology. 1999; 8(4): 211.

[93] Aijun Lu. A case of papules caused by clozapine. Journal of Clinical Psychiatry. 2014; 24(6):430

[94] Yongping Liu, Shilin Tang. A case report of allergic dermatitis caused by risperidone. Public Medical Forum Magazine. 2013; 17(1):74.

[95] Qingyun Liu, Haiying Zhao. Two cases of dermatitis caused by haloperidol. Shandong Archives of Psychiatry. 1996; (2):49.

[96] Linlin Liu, Gang Li, Ying Wang. A case report of cholinergic urticaria caused by quetiapine. J Clin Psychiatry. 2011; 21(1):20.

[97] Junlin Liu, Fengzhen Liu, Shufang Wang. A case report of morbilliform erythema caused by risperidone. Strait Pharm J. 2003; 15(1): 71.

[98] Jinnian Liu. Two cases of epidermolysis bullosa caused by clozapine. Journal of Qiqihar Medical College. 2002; 23(1):120.

[99] Hengwei Liu. A case report of severe erythema multiforme and priapism caused by clozapine. Handan Medical Journal. 1997; 10(4):299.

[100] Haiyan Liu, Chunlian Shi, Xiaonan Liu, Yunfang Liu. Epidermolysis bullosa induced by sulpiride. ADR J. 2001; (4):252.

[101] Aiqin Liu, Shubo Lv, Yanfei Xing. A case report of rash caused by risperidone. Journal of Psychiatry. 2008; 21 (1):73.

[102] Zhuxin Lin. A case report of exfoliative dermatitis caused by clozapine. Arch Psychiatry. 1995; 7(4):252.

[103] Yunquan Li, Shikang Xiao. A case report of died drug eruption with agranulocytosis caused by clozapine. Chongqing Med. 1994; 23(3):192.

[104] Xiaoxu Li, Fengmei Li. A case report of exfoliative dermatitis caused by perphenazine. Shandong Archives of Psychiatry. 2002; 15(1):13.

[105] Wenxia Li. A case report of skin rash and arthritis caused by clozapine. Shandong Archives of Psychiatry. 1989; (2):58.

[106] Shiping Li, Ximin Yang, Junyan Han. A case of drug eruption and abnormal lymphoid tissue increased significantly caused by clozapine. Shaanxi Medical Journal. 1995; 24(5):319-320.

[107] Qingfang Li, Wanping Zhu. A case report of febrile rash caused by sulpiride. Sichuan Mental Health. 1996; 9(1):27.

[108] Ping Li. A case report of drug eruption of erythema multiforme caused by sulpiride. China Aerospace Industry Medicine. 2001; 3(1): 51.

[109] Ling Li. A case report of vesicular drug eruption caused by perphenazine. Shandong Archives of Psychiatry. 1997; (03):26.

[110] Jianfen Li. Two cases of exfoliative dermatitis caused by chlorpromazine. Journal of Clinical Psychosomatic Diseases. 2005; 11(3): 227.

[111] Yanyan Jiang, Liping Ni, Lingling Wu. A case report of rash caused by risperidone. Sichuan Mental Health. 2009; 22(3):153.

[112] Xiaohong Hu. A case report of skin rash caused by sulpiride. J China Hwalth Care & nutrition. 2013; (10):822.

[113] Manji Hu, Peng Wang, Ming Sun, Qun Li. Urticaria caused by amisulpride: a case report. J Chinese Journal of Psychiatry. 2012; 45(4): 222.

[114] Fusheng Hu. A case report of papuloid skin lesions caused by long-term use of risperidone. Journal of Zhongyuan Psychiatric medicine. 2000; 6(3):148.

[115] Jiakun Hong, Yanbing Sun, Xiaobing Liu. A case report of drug-induced dermatitis caused by ziprasidone. Sichuan Mental Health. 2022; 35(04):370-372.

[116] Guoqi He. Three cases of allergic dermatitis caused by quetiapine fumarate. Herald Med. 2008; 27(3):348.

[117] Zhengmei Guo, Xin Jin. A case report of allergic dermatitis caused by oral quetiapine fumarate. World Latest Medicine Information. 2016; 16(73):191.

[118] Yuhua Guo. A case report of drug eruption caused by Risperdal. Medical Journal of Chinese Civil Administration. 2001; 13(5):291.

[119] Yingli Guo. Three cases of epidermolysis bullosa caused by sulpiride infusion in patients with mental disorders. Journal of Clinical Psychosomatic Diseases. 2016; 22(5):162-163.

[120] Weiqiang Gu, Weiguo Zhu, Chune Fu, Ting Ye. A case report of exfoliative dermatitis caused by chlorpromazine. Med J Chin People Health. 2008; 20(17):1977.

[121] Weiqiang Gu. A case report of skin rash caused by clozapine. Med J Chin People Health. 2009; 21(23):3078.

[122] Mingzhi Gu, Xinsheng Peng. A case report of systemic rose papules caused by sulpiride. Sichuan Mental Health. 1994; 7(2):90.

[123] Bingzhu Gao, Chunying Chen. Clinical management of bullous drug eruption caused by clozapine. Strait Pharm J. 1997; (S1):125-126.

[124] Zhiping Fang. A case report of exfoliative dermatitis caused by risperidone. Journal of Clinical Psychiatry. 2008; 18(5) 297.

[125] Xiaoting Fang, Ying Wei. A case report of erythema multiforme caused by olanzapine. Neural Injury and Functional Reconstruction. 2016; 11(2):190.

[126] Ke Du, Kui Jin. A case report of skin damage caused by tiapride. Chin J Nervous Mental Dis.2002; 28(3):240.

[127] Yi Dong. A case report of exfoliative dermatitis caused by clozapine. Chinese Journal of Pharmacoepidemiology. 1996; 5(2):73.

[128] Jing Deng, Yuhan Ma. A case report of allergy caused by clozapine. Chinese Journal of Medicine. 2001; 36(3):17.

[129] Wenjun Shan, Hongzhang Huang, Shujun Cheng. A case report of allergy in patient with verruca vulgaris caused by chlorpromazine treatment of local block. Clin J Med Offic. 2002; 30(2):6.

[130] Licheng Chen, Xiaowen Liu. A case of child with transient miliary rash caused by low dose risperidone. Chinese Journal of Pharmacoepidemiology. 2003; 12(1):29.

[131] Dayong Chen, Lian Wang, Shumin Zhong. A case report of death caused by chlorpromazine hydrochloride erythema multiforme. Journal of Gannan Medical College. 1982; (0):84-85.

[132] Xiuying Cao. A case of nursing experience of exfoliative dermatitis caused by clozapine. Med J Chin People Health. 2009; 21(22): 2873.

[133] Hengbing, Chong Yao. A case report of severe agranulocytosis with systemic rash caused by risperidone. Chinese Journal of Clinical Pharmacy. 2013; 22(5):318.

[134] Zhengrong Ang, Yanpu Zhou, Qingsong Ji. A case report of drug eruption and disorientation caused by clozapine. Chin J Nervous Mental Dis. 2001; 27(4):272.

[135] Zhengrong Ang, Baodong Han, Jinling Zhang. A case report of rash caused by aripiprazole. J Clin Psychol Med. 2008; 18 (2):76.

[136] Jiling Wang. A case report of allergic rash caused by clozapine. Shandong Archives of Psychiatry. 1988; (1):69.

[137] Ruiming Zhu. Allergic to multiple psychotropic drugs. Sichuan Mental Health. 1996; (4):261-262.

[138] Chunyang Li, Peixian Lu. A case report of cross allergy induced by antipsychotic drugs. Sichuan Mental Health. 2001; 14(4):195.

[139] Song He. Seven cases of nursing of exfoliative dermatitis caused by chlorpromazine. Heilongjiang Medicine and Pharmacy. 2002; 25(2):100.
